# Stereo Direct Sparse Visual–Inertial Odometry with Efficient Second-Order Minimization

**DOI:** 10.3390/s25154852

**Published:** 2025-08-07

**Authors:** Chenhui Fu, Jiangang Lu

**Affiliations:** State Key Laboratory of Industrial Control Technology, College of Control Science and Engineering, Zhejiang University, Hangzhou 310027, China; chfu@zju.edu.cn

**Keywords:** direct sparse odometry, efficient second-order minimization, marginalization, sliding window optimization

## Abstract

Visual–inertial odometry (VIO) is the primary supporting technology for autonomous systems, but it faces three major challenges: initialization sensitivity, dynamic illumination, and multi-sensor fusion. In order to overcome these challenges, this paper proposes stereo direct sparse visual–inertial odometry with efficient second-order minimization. It is entirely implemented using the direct method, which includes a depth initialization module based on visual–inertial alignment, a stereo image tracking module, and a marginalization module. Inertial measurement unit (IMU) data is first aligned with a stereo image to initialize the system effectively. Then, based on the efficient second-order minimization (ESM) algorithm, the photometric error and the inertial error are minimized to jointly optimize camera poses and sparse scene geometry. IMU information is accumulated between several frames using measurement preintegration and is inserted into the optimization as an additional constraint between keyframes. A marginalization module is added to reduce the computation complexity of the optimization and maintain the information about the previous states. The proposed system is evaluated on the KITTI visual odometry benchmark and the EuRoC dataset. The experimental results demonstrate that the proposed system achieves state-of-the-art performance in terms of accuracy and robustness.

## 1. Introduction

The ability to achieve robust and accurate ego-motion estimation is critical for autonomous systems operating in complex environments. This requirement spans a variety of applications, from micro-aerial vehicles (MAVs) conducting search-and-rescue missions in degraded visual conditions to augmented reality (AR) devices that demand millimeter-level tracking accuracy in cluttered indoor spaces. VIO, which synergistically combines camera imagery with IMU data, has emerged as the predominant approach for six-degree-of-freedom (6-DOF) state estimation in GPS-denied environments. While conventional feature-based VIO systems have demonstrated remarkable performance in structured scenarios, their dependence on explicit feature detection and matching renders them brittle in real-world conditions such as motion blur, low texture, and low illumination.

Direct sparse odometry approaches, which optimize motion parameters directly on raw pixel intensities, present a promising alternative. These methods circumvent the limitations of feature extraction, allowing them to leverage information from low-texture regions. By being tightly coupled with high-frequency IMU measurements through preintegration, direct sparse VIO systems hold the potential to achieve exceptional robustness in challenging scenarios.

Despite these theoretical advantages, practical implementations of direct sparse VIO face three key challenges:

1. Initialization Sensitivity: Joint optimization of visual–inertial parameters requires accurate initial estimates for scale, gravity direction, and sensor biases. Current direct sparse VIO systems mostly rely on specific initialization motions (e.g., slow translation) and are prone to divergence when subjected to aggressive initial maneuvers or degenerate motions.
2. Dynamic Illumination: Direct methods rely on the assumption of photometric consistency, which makes them vulnerable to errors caused by dynamic illumination. This limitation is particularly critical, as real-world environments often experience significant brightness variations.
3. Multi-Sensor Fusion: The disparate temporal characteristics of visual and inertial sensors result in complex error propagation. Existing architectures typically either oversimplify IMU dynamics or suffer from latency due to suboptimal sensor fusion strategies.

**Figure 1:**
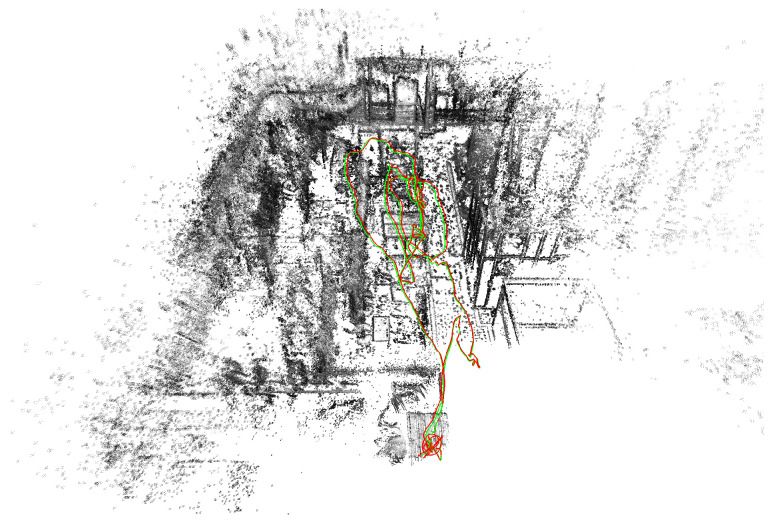
Example of stereo direct sparse visual–inertial odometry. The green line represents the ground-truth trajectory, and the red line is the estimated trajectory.

This paper proposes stereo direct sparse visual–inertial odometry (SDS-VIO) that addresses the aforementioned limitations through three key innovations (Figure 1). First, we present a visual–inertial initialization strategy that integrates IMU preintegration uncertainty with a stereo image, enabling reliable state estimation even under arbitrary initial motions. This approach eliminates the need for restrictive initialization procedures. Second, we incorporate the efficient second-order minimization (ESM) algorithm into the direct image alignment process. By using the second-order Taylor expansion for the photometric error and the first-order expansion for the Jacobian, our method achieves more efficient and accurate optimization. Finally, an adaptive tracking ratio is defined as the quotient between the number of tracked points and the number of selected points across all keyframes in the sliding window. This adaptive keyframe selection strategy enhances both the efficiency and robustness of the system.

The remainder of this paper is organized as follows: Section 2 reviews related work in visual odometry and VIO systems. Section 3 details the proposed SDS-VIO system. Section 4 describes the experimental setup and comparative analysis. Section 5 summarizes the results and future directions.

## 2. Related Work

The first real-time visual odometry (VO) system was proposed by Davison [1] around 2007, and it used a monocular camera to estimate camera motion and construct a persistent map of scene landmarks. Since their inception, VO algorithms have been broadly categorized into two axes: direct vs. indirect and dense vs. sparse.

Early VO/SLAM systems were predominantly indirect, partly due to the need for loop closure schemes in full-fledged SLAM systems, which often relied on feature descriptors [2]. Henry et al. [3] proposed a vision-based method for mobile robot localization and mapping using the SIFT for feature extraction. Among these systems, ORB-SLAM3 [4] emerged as a reference implementation of indirect approaches owing to its superior accuracy and versatility. Shen and Kong [5] utilized the Mixer MLP structure for tracking feature points, achieving high-quality matching in low-texture scenes.

Direct methods, on the other hand, recover motion parameters directly from images by minimizing photometric error based on the brightness constancy assumption [6,7,8]. Qu et al. [9] adopted the inverse compositional alignment approach to track new images with regard to the entire window and parallelized their system to effectively utilize computational resources. Wang et al. [10] presented a tightly coupled approach combining cameras, IMU and GNSS for globally drift-free and locally accurate state estimation. A direct sparse monocular VIO system was proposed by Zhang and Liu [11] based on adaptive direct motion refinement and photometric inertial bundle adjustment. DM-VIO [12] adopts delayed marginalization to address slow initialization and improve the scale estimation.

Dense methods reconstruct the entire image, using all pixels, while sparse methods only use and reconstruct a selected set of independent points. DTAM [13] is a real-time camera tracking and reconstruction system that relies on dense, per-pixel methods instead of feature extraction. Engel et al. [14] built large-scale consistent maps with highly accurate pose estimation based on an appearance-only loop detection algorithm. Gutierrez-Gomez et al. [15] minimized both photometric and geometric errors to estimate the camera motion between frames. The geometric error was parameterized by the inverse depth which translated into a better fit of its distribution to the cost functions.

However, most existing dense approaches neglect or approximate correlations between geometry parameters, along with the addition of geometric priors, making real-time statistically consistent joint optimization challenging. Additionally, as the map size grows, maintaining a dense map becomes prohibitively expensive. Forster et al. [16] used direct methods to track and triangle pixels that are characterized by high gradients, but relied on proven feature-based methods for joint optimization of structure and motion. Mourikis et al. [17] presented a measurement model that expresses geometric constraints without including 3D feature positions in the state vector. Geneva et al. [18] combined sparse visual features with inertial data in a filter-based framework, enabling efficient and lightweight state estimation, emphasizing computational efficiency and robustness in dynamic environments.

## 3. System Overview

**Figure 2:**
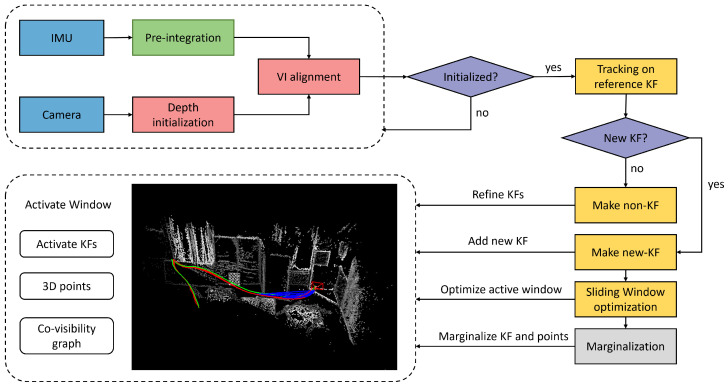
Overview of the SDS-VIO system, which mainly consists of a visual–inertial depth initialization module, a direct image alignment tracking and optimization module and a marginalization module.

The overall structure of the proposed SDS-VIO system is shown in Figure 2. It incorporates a depth initialization module, a stereo image tracking module and a marginalization module. Different from conventional random scale initialization, the system employs two-stage initialization (Section 3.4): first, depth is estimated through spatial static stereo matching, followed by visual–inertial measurement alignment. Building on direct image alignment, new stereo frames (Section 3.2) and IMU measurements (Section 3.3) undergo coarse-to-fine tracking relative to reference keyframes. The obtained pose estimation subsequently refines the depth of recently selected points. When the number of active points falls below an adaptive ratio, the system adds new keyframes to the active window (Section 3.5). For all keyframes within the window, a visual–inertial bundle adjustment is performed, optimizing their geometry, poses, affine brightness parameters, and IMU biases and velocities. To maintain the sliding window size, old keyframes and 3D points are marginalized out using the Schur complement (Section 3.6) to ensure system consistency.

### 3.1. Notation

Throughout this paper, light lower-case letters represent Wscalars (c), and bold lower-case letters represent vectors (t). Matrices are represented by bold upper-case letters (R), and functions are represented by light upper-case letters (E).

The camera intrinsic matrix is denoted as K. Camera poses are represented by matrices of the special Euclidean group $T_{i}\in SE\left(3\right)$, which transform a 3D coordinate from the camera frame to the world frame. The relative pose between two cameras is denoted as $T_{ij}$, which transforms a 3D coordinate from the *i*-th camera frame to the *j*-th camera frame.

Any 3D point $p={\left(X,Y,Z\right)}^{⊤}$ in the camera frame can be mapped to a pixel coordinate $u={\left(u,v\right)}^{⊤}$ via the projection function $\Pi_{K}:R^{3}\to R^{2}$, where

(1) $$u=\Pi_{K}\left(p\right)={\left(f_{x}\frac{X}{Z}+c_{x},f_{y}\frac{Y}{Z}+c_{y}\right)}^{⊤}.$$

Similarly, given a pixel coordinate u and its inverse depth ρ, the 3D point coordinate can be obtained via the back-projection function $\Pi_{K}^{-1}$ as

(2) $$p=\Pi_{K}^{-1}\left(u,\rho \right)={\left(\frac{u-c_{x}}{f_{x}\rho },\frac{v-c_{y}}{f_{y}\rho },\frac{1}{\rho }\right)}^{⊤}.$$

The inverse depth parameterization has been demonstrated to be advantageous when errors in images are modeled as Gaussian distributions [19]. By this, this paper uses the inverse depth and its pixel coordinate to represent a 3D point.

Similar to [6], we formulate motion estimation as an optimization problem that minimizes an error function. Specially, the re-projection process is mathematically modeled as

(3) $$p^{\prime }=W\left(p,\xi \right)=\Pi_{K}\left(\exp\left(\xi^{\wedge }\right)\Pi_{K}^{-1}\left(p,\rho \right)\right),$$

where W(·) denotes the warping function that maps the pixel coordinate p in the reference frame to the pixel coordinate $p^{\prime }$ in the target frame; ξ∈se(3) represents the camera posture parameters in the Lie algebra associated with the relative transformation between the two frames. Here, we omit the conversion from non-homogeneous coordinates to homogeneous coordinates.

### 3.2. Photometric Error

In this paper, the target frame $I_{j}$ and reference frame $I_{i}$ are treated as temporal multi-view stereo, while the stereo pair frames are treated as spatial static stereo.

Temporal Multi-View Stereo. Each residual from temporal multi-view stereo is defined as

(4) $$r_{k}^{t}=I_{j}\left[p^{\prime }\right]-b_{j}-\frac{t_{j}e^{a_{j}}}{t_{i}e^{a_{i}}}\left(I_{i}\left[p\right]-b_{i}\right),$$

where $t_{i}$ and $t_{j}$ are the exposure times, $a_{i}$, $b_{i}$, $a_{j}$, and $b_{j}$ are the coefficients to correct for affine illumination changes, and $I_{i}$ and $I_{j}$ are images of respective frames.

For image alignment tasks, traditional approaches such as the forward compositional (FC) and inverse compositional (IC) algorithms have inherent limitations. The FC method requires re-computing image gradients at each iteration, which introduces significant computational overhead. Conversely, the IC method avoids this by assuming a fixed gradient on the reference image, but this assumption often breaks down under varying illumination or geometric transformations, leading to decreased robustness and slower convergence. To address these issues, the ESM algorithm combines the advantages of both FC and IC by symmetrizing the update rule and averaging the image gradients from both frames, resulting in a more accurate approximation of the cost function’s curvature. This leads to faster and more stable convergence, particularly under challenging photometric conditions such as affine illumination changes.

Using the ESM algorithm, the Jacobian of temporal stereo is defined as

(5) $$J_{k}=\left[\frac{1}{2}\left({\left.\frac{\partial I_{j}}{\partial p^{\prime }}\right|}_{\xi }+{\left.\frac{\partial I_{i}}{\partial p^{\prime }}\right|}_{\xi ⊞\delta \xi }\right)\frac{\partial p^{\prime }\left(\xi ⊞\delta \xi \right)}{\partial \delta \xi_{geo}},\frac{\partial r_{k}\left(\xi ⊞\delta \xi \right)}{\partial \delta \xi_{photo}}\right].$$

Formally, the photometric error of a point $p\in N_{p}$ using ESM is defined as follows:

(6) $$E_{ij}:=\sum_{p\in N_{p}}w_{p}∥I_{j}\left[p^{\prime }\right]-b_{j}-\frac{t_{j}e^{a_{j}}}{t_{i}e^{a_{i}}}\left(I_{i}\left[p\right]-b_{i}\right)+J▵\xi {∥}_{\gamma },$$

where $N_{p}$ is a small set of pixels around the point *p*, γ is the Huber norm, and $w_{p}$ is a gradient-dependent weighting.

Spatial Static Stereo. For stereo pair frames, the residual is modified to

(7) $$r_{k}^{s}=I_{i}^{R}\left[p^{\prime }\left(T_{ji},d,c\right)\right]-b_{i}^{R}-\frac{t_{j}e^{a_{j}}}{t_{i}e^{a_{i}}}\left(I_{i}\left[p\right]-b_{i}^{L}\right).$$

The Jacobian of static stereo has fewer geometric parameters $\xi_{geo}=\left(d,c\right)$, because the relative transformation between the two cameras $T_{ji}$ is fixed. Therefore, it will not be optimized in the window optimization.

With that, the error function can be formulated as

(8) $$E=\sum_{i\in F}\sum_{p\in P_{i}}\left(\sum_{j\in obs(p)}E_{ij}+\alpha E_{is}\right),$$

where F is a set of keyframes that we are optimizing, $P_{i}$ is a sparse set of points in keyframe *i*, and obs(p) is a set of observations of the same point in other keyframes. The error $E_{is}$ belongs to the static stereo residuals.

### 3.3. Inertial Error

The proposed method establishes an inertial measurement error function derived from gyroscopic angular velocity and accelerometric linear acceleration data. Through the IMU preintegration approach, we formulate a unified inertial measurement constraint that characterizes the relative pose transformation between consecutive visual observation frames.

For two states $s_{i}$ and $s_{j}$, and IMU measurements $a_{i,j}$ and $\omega_{i,j}$ between two images, we obtain a prediction ${\hat{s}}_{j}$ as well as an associated covariance matrix ${\hat{\Sigma }}_{s,j}$. The corresponding error function is defined as

(9) $$E_{inertial}\left(s_{i},s_{j}\right):={\left(s_{j}⊟{\hat{s}}_{j}\right)}^{⊤}{\hat{\Sigma }}_{s,j}^{-1}\left(s_{j}⊟{\hat{s}}_{j}\right),$$

where the operator ⊟ applies $\xi_{j}⊞{\left(\hat{\xi_{j}}\right)}^{-1}$ for poses and a normal subtraction for other components.

### 3.4. Initialization and Tracking

We estimate the camera pose by minimizing the total error between the target frame and the reference frame, defined as

(10) $$E_{total}=E_{photo}+\lambda E_{inertial},$$

which consists of a photometric error term $E_{photo}$, an inertial error term $E_{inertial}$ and a coupling factor λ.

To initialize the system, the inverse depths of points in the first frame are required. Unlike previous monocular direct VO approaches that typically initialize using random depth values [6], this paper uses static stereo matching to estimate a sparse depth map for the first frame. Since the affine brightness transfer factors between the stereo image pair are unknown at this stage, correspondences are searched along the horizontal epipolar line using the NCC over a 3×5 patch, and are accepted only if the NCC score exceeds 0.95. Meanwhile, IMU measurements are preintegrated following the on-manifold model [20] to compute the initial gravity direction and provide motion constraints by averaging up to 40 accelerometer measurements, yielding a reliable estimate even under high acceleration. The stereo-derived depth and IMU information are then jointly used to compute the initial camera pose, velocity, and gravity-aligned reference frame.

The initial inverse depths obtained from the stereo are not treated as fixed values. Similar to DSO, they are jointly optimized along with camera poses and velocities within a sliding window. Preintegrated IMU measurements are incorporated as residuals, and weighted by their covariances, enabling tight visual–inertial coupling. This joint optimization naturally refines initial uncertainties, without explicit thresholding on depth confidence.

Each time a new stereo frame is fed into the system, direct image alignment is used to track it. All the points inside the active window are projected into the new frame. Then the pose of the new frame is optimized by minimizing the error function. The optimization is performed using the Gauss–Newton method on an image pyramid in a coarse-to-fine manner. If the residual exceeds a predefined level, scaled relative to a minimum threshold specific to each image pyramid level, we reject the frame. The threshold is set empirically as 1.5× the minimum residual, which offers a good balance and has been used consistently.

### 3.5. Sliding Window Optimization

Our system maintains a sliding window of *N* keyframes $K=K_{1},\ldots ,K_{N}$. Each keyframe $K_{i}$ is associated with a Gaussian pyramid of images $I_{i}=I_{i}^{0},\ldots ,I_{i}^{P}$, a set of affine brightness parameters $a_{i}={\left(a_{i},b_{i}\right)}^{⊤}$, a camera pose $T_{i}^{W}\in \mathrm{SE}\left(3\right)$ with regard to the world frame W, a set of $m_{k}$ points parameterized by inverse depth $\rho_{i,p}$ hosted in the keyframe, the current IMU bias $b_{i}\in R^{6}$, and the velocity $v_{i}\in R^{3}$.

We compute the Gauss–Newton as

(11) $$H=J^{⊤}WJ,\;b=-J^{⊤}Wr,$$

where $W\in R^{n\times n}$ is the diagonal matrix containing the weights, $r\in R^{n}$ is the stacked residual vector, and $J\in R^{n\times d}$ is the Jacobian of r.

Since the visual error term $E_{photo}$ and the inertial error term $E_{inertial}$ are independent, the Hessian matrix H and the residual vector b can be divided into two parts:

(12) $$H=H_{photo}+H_{inertial},\;b=b_{photo}+b_{inertial}.$$

The formulation of inertial error residuals is inherently expressed within the body-attached sensor coordinate system, whereas the joint state estimation process occurs within a globally referenced spatial framework. To reconcile this reference discrepancy, we introduce a Jacobian operator $J_{wi}$ that propagates infinitesimal variations from the local inertial measurements to the global state perturbations. As a result, the inertial residuals lead to

(13) $$H_{inertial}=J_{wi}^{⊤}H_{inertial}^{\prime }J_{wi},\;b_{inertial}=J_{wi}^{⊤}b_{inertial}^{\prime }.$$

A keyframe is only needed when the current image cannot be reliably tracked with respect to the sliding window. If a sufficient number of points from the local map can be successfully projected into the image, we can simply continue using the existing keyframes. This approach prevents the addition of new keyframes that provide minimal contribution to frame tracking. Quantitatively, we define the tracking ratio *Q* as the ratio between the number of tracked points and selected points from all keyframes in the window. A new keyframe is created if *Q* falls below a threshold $Q_{min}$.

### 3.6. Marginalization

**Figure 3:**
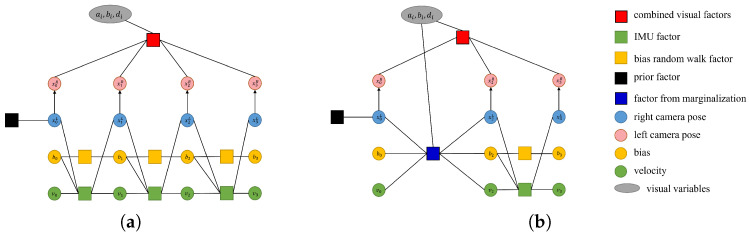
Factor graphs for the visual–inertial joint optimization before (**a**) and after (**b**) the marginalization of a keyframe.

With each iteration, the number of states and the computational complexity increase quadratically. To limit this, marginalization is applied to preserve useful information. The procedure converts previous measurements into a prior term, maintaining past information. Visual factor marginalization follows the approach in [21], where residual terms affecting sparsity are discarded, and all keyframe points are marginalized by marginalizing the keyframe itself. Figure 3 shows how marginalization changes the factor graph. The states to be marginalized are denoted as $X_{m}$, and the remaining states are denoted as $X_{r}$. Marginalizing the states reduces the size of optimization problem while updating matrices H and b. After reordering the states, the optimization formulation is updated as follows:

(14) $$\left[\begin{array}{cc} H_{mm} & H_{mr}\\ H_{rm} & H_{rr} \end{array}\right]\left[\begin{array}{c} \delta X_{m}\\ \delta X_{r} \end{array}\right]=\left[\begin{array}{c} b_{m}\\ b_{r} \end{array}\right].$$

The marginalization is carried out using the Schur complement as

(15) $$\underset{H_{p}}{\underset{︸}{\left(H_{rr}-H_{rm}H_{mm}^{-1}H_{mr}\right)}}\delta X_{r}=\underset{b_{p}}{\underset{︸}{b_{r}-H_{rm}H_{mm}^{-1}b_{m}}}.$$

We compute a new prior term $H_{p}$ and $b_{p}$ for the remaining states, incorporating the information from marginalized states without loss. Specifically, our system maintains seven spatial camera frames, and when a new keyframe is added, we marginalize out the visual and inertial factors related to the states of the first frame.

## 4. Evaluation

We evaluate the proposed method on two established benchmarks: the KITTI visual odometry benchmark [22] and the EuRoC dataset [23]. In each experiment, the number of active points and keyframes retained in the local map is set to 2000 and 7, respectively. A constant coupling factor of α=3 is used throughout the tests.

### 4.1. KITTI Visual Odometry Benchmark

The KITTI visual odometry benchmark consists of 22 sequences, all collected from a moving car. The datasets primarily feature street scenes with dynamic objects. Among the 22 sequences, ground-truth 6D poses are available only for the first 11. Therefore, the evaluation is primarily conducted on these first 11 sequences.

**Figure 4:**
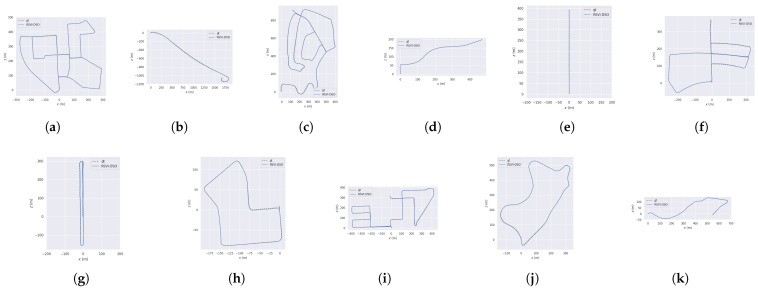
Trajectory comparison with ground truth across all train sequences (00–10) in the KITTI dataset: (**a**) sequence 00; (**b**) sequence 01; (**c**) sequence 02; (**d**) sequence 03; (**e**) sequence 04; (**f**) sequence 05; (**g**) sequence 06; (**h**) sequence 07; (**i**) sequence 08; (**j**) sequence 09; (**k**) sequence 10.

Figure 4 shows the trajectories generated by SDS-VIO across all test sequences in the KITTI benchmark compared with the ground truth. Among the paths, sequences 00, 02, 05, 08, 09 and 10 represent long sequences in large environments, while sequences 06, 07 and 09 are relatively short with significant rotation. The remaining sequences are short and relatively straight. It can be seen that SDS-VIO performs well in all cases without distinct scale drift.

**Figure 5:**
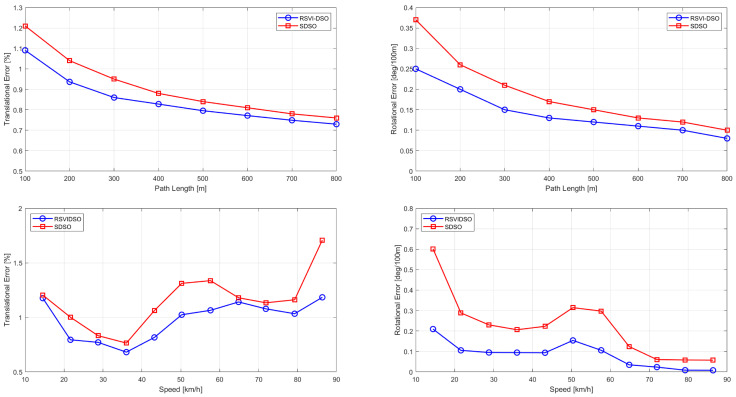
Example of average translation and rotation errors with respect to the path length (top two) and moving speed (bottom two) on sequence 06.

In Figure 5, we compare SDS-VIO with SDSO [8] in terms of average translation and rotation errors. The errors are calculated relative to the path length and moving speed. The results demonstrate that our method outperforms SDSO in all cases. Specifically, SDS-VIO exhibits strong robustness and accuracy across varying moving speeds and path lengths.

We compared our method to SDSO and R-SDSO, which are currently the state-of-the-art stereo direct VO methods. The results are shown in Table 1. The results for R-SDSO are taken from [24], while those for SDSO are obtained by running their code with default settings. It can be observed that the proposed method generally outperforms SDSO. Compared to R-SDSO, our method achieves a better performance in most sequences, although the translational errors show slight variation. This may be attributed to the relatively low frame rate of the dataset, which reduces the effectiveness of IMU measurements.

### 4.2. EuRoC Dataset

**Figure 6:**
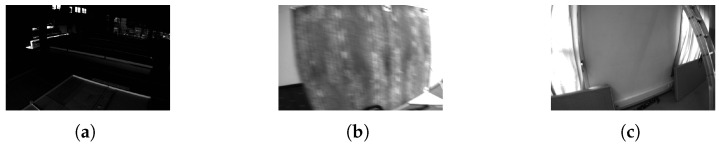
Example images from the EuRoC dataset: (**a**) low illumination, (**b**) strong motion blur, (**c**) low texture features.

The EuRoC dataset provides high-quality data collected from MAVs in two environments: an industrial machine hall and a Vicon room. As shown in Figure 6, the Euroc dataset poses challenges due to low illumination, strong motion blur and low texture features. To ensure an accurate evaluation, each method runs 10 times for each sequence in the dataset.

Table 2 shows the Absolute Trajectory Error (ATE) comparison to several other methods. The “X” that indicates the method failed to track the sequence. The results for OKVIS [25] and VI-DSO are quoted from [21], while the results of BASALT and VINS-Fusion are quoted from [26,27]. Compared to other methods, our method obviously outperforms them in terms of RMSE across most sequences. In more challenging sequences, such as V2_03_difficult, our method continues to demonstrate robust performance, while BASALT and OKVIS were unable to track this sequence. Note that the Vicon room sequences (V*) are executed in a small room with many looped motions where the loop closures in SLAM systems significantly improve the performance. Overall, the results demonstrate that SDS-VIO consistently delivers superior performance across all evaluated sequences.

**Figure 7:**
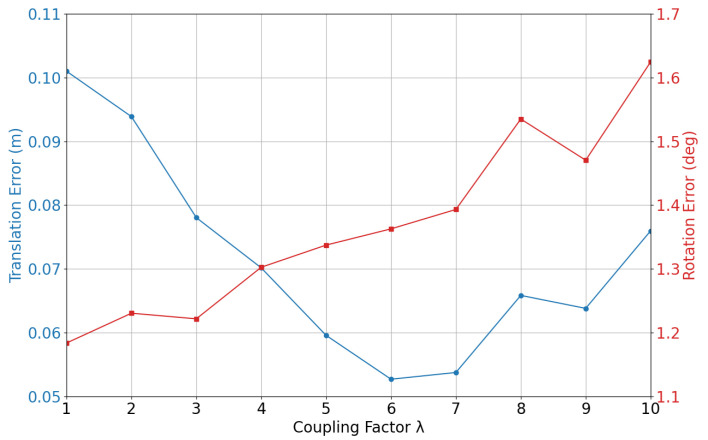
Average translation and rotation errors on sequence MH_03_medium for different inertial coupling factors.

Additionally, we test the influence of the inertial coupling factor on the example sequence V1_03_difficult. The translation and rotation errors are shown in Figure 7. As λ increases, the rotation error gradually increases, which indicates that the system is more sensitive to inertial measurements. However, the translation error shows a slight decrease at first and then increases, indicating that the system is able to utilize inertial measurements to improve tracking performance up to a certain point. The results suggest that a moderate coupling factor (λ=6) is beneficial to achieve a balance between precision and robustness.

### 4.3. Speed and Accuracy

We benchmark SDS-VIO, SDSO and VINS-Fusion with single threaded settings on a desktop computer with an Intel i5-14600K CPU and 32 GB RAM. We run both systems on the V1_03_difficult sequence from the EuRoc dataset and average timing results over several runs. Additionally, to examine the effectiveness of the ESM algorithm, tracking without the ESM algorithm is also added for comparison. We again use the default settings for both VINS-Fusion and SDSO (with 7 keyframes and 2000 points max) and do not enforce real-time execution (no skipping frames). Note that it is difficult to ensure a completely fair comparison, as each system uses slightly different window sizes, pyramid levels, number of iterations, and other hyper-parameters that may affect its performance.

Runtime results are shown in Table 3. SDS-VIO with ESM achieves the best performance, with an average time of 42.67 ms per frame while tracking, which is significantly faster than SDSO and VINS-Fusion. The results also show that the ESM algorithm is more efficient than the FC algorithm, as it requires less time to compute the Jacobian and residuals. The accuracy of SDS-VIO is also better than SDSO and R-SDSO in terms of translation and rotation errors, demonstrating that the proposed method can achieve real-time performance while maintaining high accuracy.

## 5. Conclusions

In this work, we propose a stereo direct sparse visual-inertial odometry (SDS-VIO) system with efficient second-order minimization for accurate real-time tracking and mapping. We detailed the technical implementation including the integration of multi-stage initialization, direct image alignment with ESM, and adaptive sliding window optimization. The superior performance of SDS-VIO is demonstrated through both qualitative and quantitative evaluations on the KITTI visual odometry benchmark and the EuRoC dataset. The results on the KITTI dataset show that SDS-VIO performs better in mean translation and rotation errors compared to R-SDSO and SDSO. Additionally, the comparison on the EuRoC dataset highlights the robustness of SDS-VIO in environments with brightness variation, motion blur and low texture features.

In future work, a database for map maintenance and the incorporation of loop closure will be considered to further improve the accuracy of SDS-VIO and extend it to be a visual–inertial fused SLAM system.

## Figures and Tables

**Table 1:** The KITTI visual odometry benchmark results.

Sequence	SDS-VIO		R-SDSO		SDSO	
	$t_{\mathrm{rel}}$	$r_{\mathrm{rel}}$	$t_{\mathrm{rel}}$	$r_{\mathrm{rel}}$	$t_{\mathrm{rel}}$	$r_{\mathrm{rel}}$
00	**0.74**	**0.27**	**0.90**	**0.30**	1.10	0.34
01	**1.63**	**0.04**	**1.53**	**0.09**	1.67	0.12
02	**0.72**	**0.23**	**0.94**	**0.25**	0.98	0.29
03	**0.91**	**0.16**	**0.93**	0.34	0.96	**0.31**
04	**0.61**	**0.11**	**0.75**	**0.15**	1.01	0.18
05	**0.62**	**0.21**	**0.96**	**0.25**	1.01	0.28
06	**0.85**	0.25	**0.88**	**0.20**	0.90	**0.21**
07	**0.75**	**0.10**	**0.83**	**0.35**	0.93	0.48
08	**1.03**	**0.13**	**1.08**	**0.26**	1.16	0.29
09	**0.91**	**0.25**	**1.17**	0.31	1.22	**0.29**
10	**0.65**	**0.07**	**0.75**	**0.29**	1.17	0.30

Comparison of accuracy on the KITTI visual odometry benchmark. $t_{rel}$ represents the translational RMSE (%), and $r_{rel}$ represents the rotational RMSE (degree per 100 m). Both values are averaged over 100 m to 800 m intervals. The best results are highlighted in bold red, while suboptimal results are displayed in bold underlined blue.

**Table 2:** The EuRoC dataset results.

Sequence	SDS-VIO	BASALT	OKVIS	VI-DSO	VINS-Fusion
MH_01_easy	**0.025**	0.070	0.230	**0.062**	0.240
MH_02_easy	**0.027**	0.060	0.150	**0.044**	0.180
MH_03_medium	**0.056**	**0.070**	0.230	0.117	0.230
MH_04_difficult	**0.028**	**0.130**	0.320	0.132	0.390
MH_05_difficult	**0.076**	**0.110**	0.360	0.121	0.190
V1_01_easy	**0.054**	**0.040**	**0.040**	0.059	0.100
V1_02_medium	**0.064**	**0.050**	0.080	0.067	0.100
V1_03_difficult	**0.085**	0.100	0.130	**0.096**	0.110
V2_01_easy	**0.052**	**0.040**	0.100	**0.040**	0.120
V2_02_medium	0.068	**0.050**	0.170	**0.062**	0.100
V2_03_difficult	**0.132**	X	X	**0.174**	0.270
Mean	**0.061**	**0.072**	0.217	0.089	0.180

The best results are highlighted in bold red, while suboptimal results are displayed in bold underlined blue. “X” indicates that the method failed to track the sequence.

**Table 3:** Speed and accuracy comparison on the V1_03_difficult sequence from the EuRoC dataset.

Method	Tracking per Frame (ms)	$t_{\mathrm{rel}}$ (m)	$r_{\mathrm{rel}}$ (deg)	RMSE (m)
SDS-VIO (ESM)	**42.670**	**0.215**	**0.220**	**0.056**
SDS-VIO (FC)	**58.250**	**0.358**	0.403	**0.080**
SDSO	79.600	0.422	**0.117**	0.115
VINS-Fusion	82.670	0.599	0.903	0.230

The best results are highlighted in bold red, while suboptimal results are displayed in bold underlined blue.

## Data Availability

The original contributions presented in this study are included in the article. Further inquiries can be directed to the authors.
